# Sound tuning of amygdala plasticity in auditory fear conditioning

**DOI:** 10.1038/srep31069

**Published:** 2016-08-04

**Authors:** Sungmo Park, Junuk Lee, Kyungjoon Park, Jeongyeon Kim, Beomjong Song, Ingie Hong, Jieun Kim, Sukwon Lee, Sukwoo Choi

**Affiliations:** 1 School of Biological Sciences, College of Natural Sciences, Seoul National University, 1 Gwanangno, Seoul 08826, Korea; 2 Center for Neuroscience and Center for Functional Connectomics, Korea Institute of Science and Technology, Seoul 136791, Korea; 3 Institute of Neuroscience, Technical University of Munich, 80333, Germany; 4 The Solomon H. Snyder Department of Neuroscience, Johns Hopkins University School of Medicine, Baltimore, Maryland 21205, USA; 5 Ewha Brain Institute, Ewha W. University, Seoul, Korea; 6 Department of Brain and Cognitive Sciences, Scranton College, Ewha W. University, Seoul, Korea; 7 Department of Neural Development and Disease, Korea Brain Research Institute, Daegu, Korea

## Abstract

Various auditory tones have been used as conditioned stimuli (CS) for fear conditioning, but researchers have largely neglected the effect that different types of auditory tones may have on fear memory processing. Here, we report that at lateral amygdala (LA) synapses (a storage site for fear memory), conditioning with different types of auditory CSs (2.8 kHz tone, white noise, FM tone) recruits distinct forms of long-term potentiation (LTP) and inserts calcium permeable AMPA receptor (CP-AMPAR) for variable periods. White noise or FM tone conditioning produced brief insertion (<6 hr after conditioning) of CP-AMPARs, whereas 2.8 kHz tone conditioning induced more persistent insertion (≥6 hr). Consistently, conditioned fear to 2.8 kHz tone but not to white noise or FM tones was erased by reconsolidation-update (which depends on the insertion of CP-AMPARs at LA synapses) when it was performed 6 hr after conditioning. Our data suggest that conditioning with different auditory CSs recruits distinct forms of LA synaptic plasticity, resulting in more malleable fear memory to some tones than to others.

Conditioned fear memory and the associated synaptic and behavioral plasticities have been extensively studied, and various auditory tones have been widely used as conditioned stimuli (CS)^1^,^2^,^3^,^4^,^5^,^6^. However, researchers have largely neglected the potential effects of different types of auditory tone used on the learning process without systematical assessments; for example, pure tones (1 to 20 kHz) and complex tones (white noise to frequency-modulated (FM) tones) have been used liberally with little justification in previous studies^3^,^7^,^8^,^9^,^10^,^11^. One of the basic assumptions underlying this trend is that fear conditioning with any one of these various tones shares the same mechanisms underlying conditioning-induced synaptic and behavioral plasticities, but this assumption has not been rigorously tested. In fact, there have been no examples in which Pavlovian conditioning of distinct sensory stimuli of the same modality (e.g., different auditory tones) produces different forms of synaptic and behavioral plasticities. Therefore, a critical question to pose would be whether different types of synaptic plasticity play distinct roles even in the memory encoding different CSs of the same modality.

The lateral amygdala is known as a storage site of conditioned fear memory^1^,^6^,^12^,^13^,^14^. Many forms of synaptic plasticity, including long-term potentiation (LTP) and conditioning-induced trafficking of AMPA receptors, have been proposed at LA synapses as a potential cellular mechanism of fear memory storage^15^,^16^,^17^. However, their precise physiological roles have not yet been characterized. Three different forms of LTP have been found at thalamic and cortical input synapses onto the LA, respectively: (1) pairing-induced LTP^17^,^18^,^19^, input timing-dependent plasticity (ITDP)^11^ and presynaptic LTP at T-LA synapses^20^,^21^,^22^; (2) pairing-induced LTP^8^, ITDP^11^ and presynaptic LTP^23^,^24^,^25^,^26^ at C-LA synapses. Most of these forms of LTP have not been rigorously tested for whether they indeed underlie conditioned fear memory, except for one form of T-LA LTP (i.e., pairing-induced LTP at T-LA synapses^17^). Additionally, a recent study has shown that fear conditioning drives trafficking of calcium permeable AMPA receptors (CP-AMPARs) onto synapses^27^. Although this trafficking has been shown to be involved in reconsolidation-update (a variation of fear extinction that induces fear memory erasure), it remains to be elucidated how this trafficking is involved in a physiological process such as fear memory consolidation. Thus, it would be highly important to dissect out the precise roles of these synaptic plasticities in the formation, maintenance or modulation of conditioned fear memory.

Reconsolidation-update is a variation of fear extinction in which conditioned and consolidated memory is reactivated by presenting one CS to conditioned animals approximately 1 hr prior to extinction training. Strikingly, reconsolidation-update produces fear memory erasure, which is evidenced by a decrease in fear relapse^28^,^29^,^30^,^31^,^32^,^33^, shedding light on a potential clinical application for treating aberrant fear-related disorders such as PTSD and phobia. Previous studies have suggested that reconsolidation-update depends on the insertion of CP-AMPARs at LA synapses^27^,^34^. Although a similar procedure has been reproduced in humans and animals^28^,^30^,^31^,^35^,^36^, some researchers have found the reconsolidation-update phenomenon difficult to reproduce^37^,^38^. To date, there have been no clear explanations for the reported discrepancies. Thus, it is important to determine what types of variables can alter the outcomes of reconsolidation-update, which depends on CP-AMPAR trafficking to LA synapses.

In the present study, we found that different auditory CSs (2.8 kHz, white noise or FM tones) recruit distinct forms of LTP, respectively. These different auditory CSs also produced synaptic insertion of CP-AMPARs for a variable period, respectively. CP-AMPARs, which were inserted after pure tone conditioning, were maintained stably (≥6 hr after conditioning), whereas complex tone conditioning produced a transient insertion (<6 hr after conditioning) of AMPA receptors. Consistent with this observation, reconsolidation-update, which requires synaptic surface expression of CP-AMPARs, was observed 6 hr after pure tone conditioning but not after complex tone conditioning.

## Results

### Fear conditioning with different auditory CSs produces pre- and postsynaptic enhancements at LA synapses

**Figure 1:**
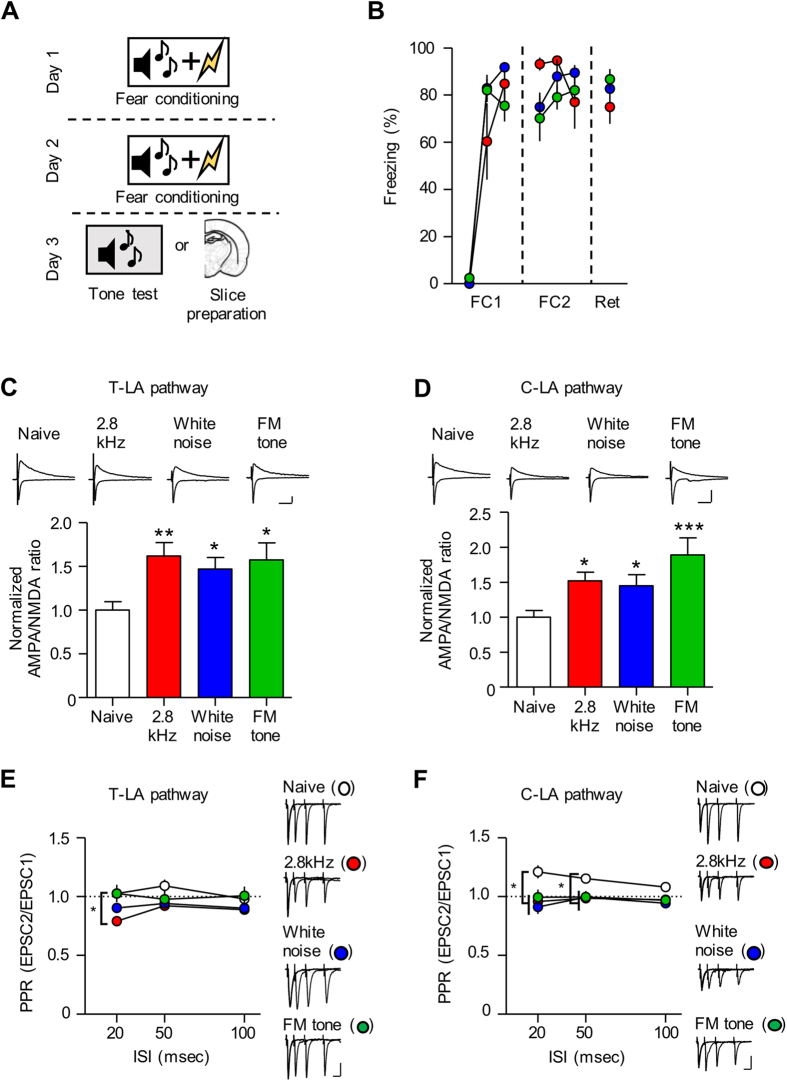
Fear conditioning with different auditory CS produces pre- and postsynaptic enhancement at LA synapses. (**A**) The behavioral procedure is shown. Fear conditioning was performed on day 1 and 2. On day 3, one group of rats was used for slice preparation, and the others were used for tone testing. (**B**) The freezing responses during fear conditioning and retention are indicated. (**C**) AMPA/NMDA EPSC ratios at T-LA synapses were expressed as a percent of those in naïve controls. AMPA/NMDA ratios in the conditioned group (2.8 kHz, white noise, FM tone) were significantly larger than those in the naïve control group (*p < 0.05, **p < 0.01 for naïve controls vs. conditioned groups, one-way ANOVA followed by Newman-Keuls post-hoc test; scale bar, 50 ms and 50 pA; white bar, naïve; red bar, 2.8 kHz; blue bar, white noise; green bar, FM tone). (**D**) AMPA/NMDA EPSC ratios at C-LA synapses were expressed as a percent of those in naïve controls. AMPA/NMDA ratios in the conditioned group (2.8 kHz, white noise, FM tone) were significantly larger than those in the naïve controls (*p < 0.05, ***p < 0.001 for naïve controls vs. conditioned groups, one-way ANOVA followed by Newman-Keuls post-hoc test; scale bar, 50 ms and 50 pA; white bar, naïve; red bar, 2.8 kHz; blue bar, white noise; green bar, FM tone). (**E**) PPR at T-LA synapses decreased only in the 2.8 kHz tone group (white circle, naïve; red circle, 2.8 kHz; blue circle, white noise; green circle, FM tone). **(F**) PPR at C-LA synapses decreased in all three conditioned groups (white circle, naïve; red circle, 2.8 kHz; blue circle, white noise; green circle, FM tone; scale bar, 20 ms and 50 pA).

We chose to use three different auditory CSs because these CSs were widely used in previous studies^3^,^7^,^8^,^10^,^11^,^38^ and because they are representatives of a wide span of auditory stimuli, including a pure tone (2.8 kHz), a broad-band mixture of pure tones (white noise), and a complex tone (frequency-modulated tone), respectively. We first characterized how conditioned fear affects the basic properties of LA synapses after conditioning with the three different types of auditory CSs (2.8 kHz tone, white noise, FM tone) (Fig. 1). To achieve stronger conditioning, we chose to repeat three CS-US pairings once a day twice (six pairings in total). We found that six pairings of CS and US produced robust freezing responses but showed no significant differences in freezing (assessed 24 hr after conditioning) among the three groups (2.8 kHz, 74.98 ± 7.12%, n = 6; white noise, 82.69 ± 2.20%, n = 8; FM tone, 86.75 ± 4.34%, n = 8; F_(2,21)_ = 1.601, *p* = 0.2277, one-way ANOVA; *p* > 0.05 for all pairs, Newman-Keuls post-hoc test) (Fig. 1B). We then assessed postsynaptic and presynaptic changes at thalamic and cortical input synapses onto the LA (thalamo-lateral amygdala (T-LA) and cortico-lateral amygdala (C-LA) synapses, respectively) after conditioning with different types of auditory CSs (2.8 kHz tone, white noise, FM tone). Postsynaptic changes after conditioning were assessed by measuring the ratio of AMPA/NMDA receptor-mediated EPSCs. At T-LA synapses, we found a significant potentiation of the ratio 24 hr after conditioning but no significant differences in the potentiation among the three groups (naïve, 1.00 ± 0.10, n = 20; 2.8 kHz, 1.62 ± 0.15, n = 14; white noise, 1.47 ± 0.13, n = 13; FM tone, 1.58 ± 0.20, n = 11; F_(3,57)_ = 5.119, *p* = 0.0034, one-way ANOVA; *p* > 0.05 for naïve controls vs. conditioned groups) (Fig. 1C). Similarly, at C-LA synapses, conditioning produced a significant potentiation of the ratio in the three groups, and we did not find any significant difference among the three groups (naïve, 1.00 ± 0.10, n = 21; 2.8 kHz, 1.52 ± 0.13, n = 8; white noise, 1.45 ± 0.16, n = 8; FM tone, 1.89 ± 0.24, n = 9; F_(3,45)_ = 7.304, *p* = 0.0005, one-way ANONA; *p* > 0.05 for naïve controls vs. conditioned groups) (Fig. 1D). Therefore, our data indicate that conditioning using the three different types of auditory CS produces enhancements in postsynaptic functions at both T-LA and C-LA synapses.

Next, we measured the paired pulse ratio (PPR) (which correlates inversely with presynaptic release probability) in response to paired stimuli at various intervals (20, 50, 100 ms) among the three groups 24 hr after conditioning. At T-LA synapses, conditioning produced a decrease in PPR at an interval of 20 ms in the 2.8 kHz group but not in the FM tone group or in the white noise group (20 ms ISI; naïve, 1.03 ± 0.05, n = 27; 2.8 kHz, 0.79 ± 0.04, n = 15; white noise, 0.90 ± 0.04, n = 18; FM tone, 1.03 ± 0.07, n = 8; F_(3.67)_ = 5.503, *p* = 0.0020, one-way ANONA; *p* < 0.05 for naïve controls vs. 2.8 kHz, Newman-Keuls post-hoc test) (Fig. 1E). At C-LA synapses, all three groups showed a decrease in PPR at intervals of 20 and 50 ms (20 ms ISI; naïve, 1.21 ± 0.06, n = 32; 2.8 kHz, 0.96 ± 0.06, n = 18; white noise, 0.91 ± 0.06, n = 17; FM tone, 1.00 ± 0.07, n = 9; F_(3,75)_ = 5.880, *p* = 0.0012, one-way ANOVA, *p* < 0.05 for naïve controls vs. the other three conditioned groups: 50 ms ISI; naïve, 1.15 ± 0.04, n = 32; 2.8 kHz, 0.99 ± 0.04, n = 18; white noise, 1.00 ± 0.05, n = 17; FM tone, 1.00 ± 0.05, n = 9; F_(3, 75)_ = 3.851, *p* = 0.0129; *p* < 0.05 for naïve controls vs. the three conditioned groups, Newman-Keuls post-hoc test) (Fig. 1F). Thus, conditioning only with the 2.8 kHz tone, but not with white noise or with the FM tone, appears to produce enhancements in presynaptic release probability at T-LA, whereas conditioning with all three different CSs seems to induce enhancements in presynaptic release probability at C-LA synapses.

Taken together, conditioning with the three different types of auditory CSs produces enhancements in both pre- and postsynaptic functions at C-LA synapses, but it only recruits postsynaptic functions at T-LA synapses, except for the 2.8 kHz tone conditioning, which also enhances presynaptic release probability at T-LA synapses.

### Fear conditioning with the different auditory CSs recruits distinct forms of LA LTP

We then asked whether mechanisms underlying synaptic potentiation are also similar among the three different groups (2.8 kHz tone, white noise and FM tone). To test this, we first determined whether conditioning with the three different auditory tones would utilize the same set of LA LTP. LA LTP has been extensively characterized, and three different forms of LTP have been found at T-LA and C-LA synapses in brain slice preparation, respectively; that is, six different forms of LA LTP in total. T-LA synapses exhibit pairing LTP, input timing dependent plasticity (ITDP) and presynaptic LTP, and C-LA synapses show pairing LTP, ITDP and Poisson LTP^8^,^11^,^17^,^18^,^19^,^20^,^21^,^22^,^23^,^24^,^25^,^26^.

**Figure 2:**
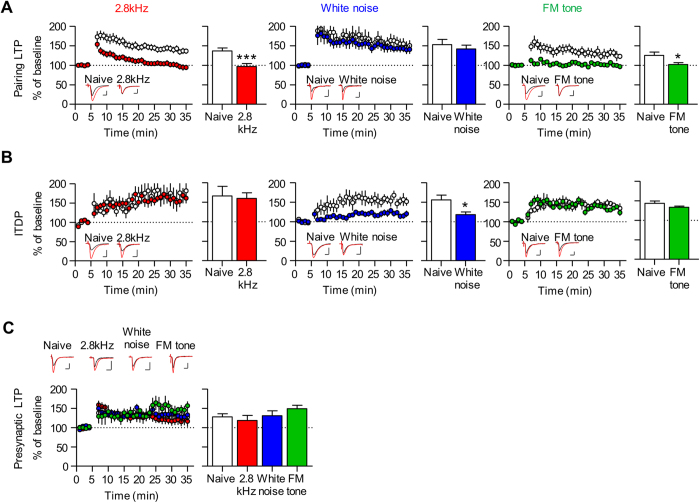
Fear conditioning with different auditory CS recruits distinct forms of LTP at T-LA synapses. (**A**) Pairing LTP was occluded in slices prepared from conditioned rats with the 2.8 kHz and FM tones but not with white noise. **Left**, pairing LTP was occluded in slices prepared from conditioned rats with 2.8 kHz (naïve, 136.77 ± 7.53%, n = 25; 2.8 kHz, 96.83 ± 7.57%, n = 24; *p* = 0.0005, unpaired *t*-test). **Middle**, pairing LTP was not occluded in slices prepared from conditioned rats with white noise (naïve, 152.77 ± 13.33%, n = 10; white noise, 141.75 ± 9.58%, n = 11; *p* = 0.5044, unpaired *t*-test). **Right**, pairing LTP was occluded in slices prepared from conditioned rats with FM tone (naïve, 125.09 ± 8.74%, n = 7; FM tone, 102.01 ± 4.70%, n = 11; *p* = 0.0217, unpaired *t*-test). (**B**) ITDP was occluded in slices prepared from conditioned rats only with white noise but not with the other two auditory stimuli. **Left**, ITPD was not occluded in slices prepared from conditioned rats with 2.8 kHz (naïve, 166.63 ± 20.28%, n = 6; 2.8 kHz, 165.89 ± 18.04%, n = 7; *p* = 0.9787, unpaired *t*-test). **Middle**, ITPD was occluded in slices prepared from conditioned rats with white noise (naïve, 155.72 ± 12.50%, n = 8; white noise, 117.99 ± 6.93%, n = 10; *p* = 0.0133, unpaired *t*-test). **Right**, ITPD was not occluded in slices prepared from conditioned rats with FM tone (naïve, 143.56 ± 5.89%, n = 7; FM tone, 133.05 ± 3.02%, n = 6; *p* = 0.1605, unpaired *t*-test). (**C**) Presynaptic LTP was not occluded in any of the three groups (naïve, 127.56 ± 8.23%, n = 18; 2.8 kHz, 118.37 ± 13.23%, n = 10; white noise, 130.76 ± 12.77%, n = 6; FM tone, 148.76 ± 8.88%, n = 5; F_(3,38)_ = 0.8482, *p* = 0.4770, one-way ANOVA; *p* > 0.05 for all pairs, Newman-Keuls post-hoc test; scale bar, 10 ms and 50 pA; *p < 0.05, ***p < 0.001, unpaired *t*-test).

To determine whether the six forms of LA LTP are differentially involved in conditioning with the three different auditory CSs, we tested whether each form of LA LTP is occluded in acute brain slices prepared from conditioned rats with the three different auditory CSs, respectively (2.8 kHz tone, white noise and FM tone). We first performed the LTP occlusion experiment in the T-LA pathway (Fig. 2). Pairing LTP was occluded in slices prepared from conditioned rats with the 2.8 kHz tone and the FM tone but not with white noise (naïve, 136.77 ± 7.53%, n = 25, 2.8 kHz, 96.83 ± 7.57%, n = 24; *p* = 0.0005, unpaired *t*-test; naïve, 152.77 ± 13.33%, n = 10, white noise, 141.75 ± 9.58%, n = 11, *p* = 0.5044, unpaired *t*-test; naïve, 125.09 ± 8.74%, n = 7, FM tone, 102.01 ± 4.70%, n = 11, *p* = 0.0217, unpaired *t*-test) (Fig. 2A). ITDP was occluded in slices prepared from conditioned rats only with white noise (Fig. 2B, middle; naïve, 155.72 ± 12.50%, n = 8, white noise, 117.99 ± 6.93%, n = 10, *p* = 0.0133, unpaired *t*-test) but not with the other two auditory CSs (Fig. 2B, left and right; left, naïve, 166.63 ± 20.28%, n = 6, 2.8 kHz, 165.89 ± 18.04%, n = 7, *p* = 0.9787, unpaired *t*-test; right, naïve, 143.56 ± 5.89%, n = 7; FM tone, 133.05 ± 3.02%, n = 6, *p* = 0.1605, unpaired *t*-test). By contrast, we failed to observe occlusion of presynaptic LTP with any of the three groups (naïve, 127.56 ± 8.23%, n = 18; 2.8 kHz, 118.37 ± 13.23%, n = 10; white noise, 130.76 ± 12.77%, n = 6; FM tone, 148.76 ± 8.88%, n = 5; F_(3,38)_ = 0.8482, *p* = 0.4770, one-way ANOVA; *p* > 0.05 for all pairs, Newman-Keuls post-hoc test) (Fig. 2C).

**Figure 3:**
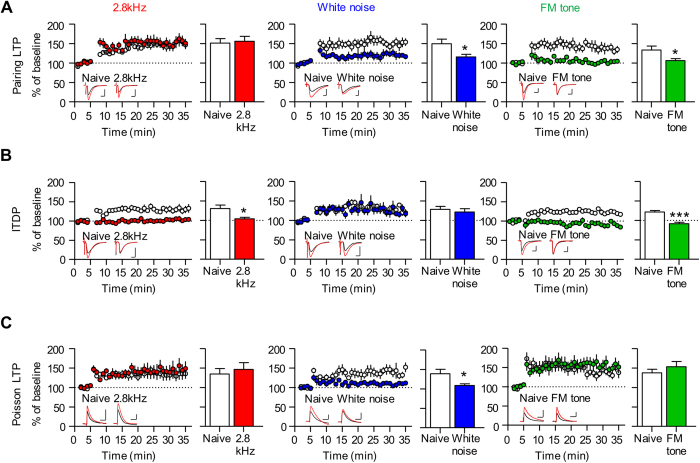
Fear conditioning with different auditory CS recruits distinct forms of LTP at C-LA synapses. (**A**) Pairing LTP was occluded in slices prepared from conditioned rats with white noise and FM tones but not with the 2.8 kHz tone. **Left**, pairing LTP was not occluded in slices prepared from conditioned rats with 2.8 kHz (naïve, 151.20 ± 11.46%, n = 11; 2.8 kHz, 155.64 ± 12.72%, n = 8; *p* = 0.8002, unpaired *t*-test). **Middle**, pairing LTP was occluded in slices prepared from condit ioned rats with white noise (naïve, 149.44 ± 11.58%, n = 6; white noise, 115.30 ± 6.86%, n = 6; *p* = 0.0294, unpaired *t*-test). **Right** pairing LTP was occluded in slices prepared from conditioned rats with FM tone (naïve, 133.36 ± 10.02%, n = 8; FM tone, 105.99 ± 5.25%, n = 9; *p* = 0.0244, unpaired *t*-test; scale bar, 10 ms and 50 pA). (**B**) ITDP was occluded in slices prepared from conditioned rats with the 2.8 kHz and FM tones but not with white noise. **Left**, ITDP was occluded in slices prepared from conditioned rats with 2.8 kHz (naïve, 130.38 ± 8.92%, n = 17; 2.8 kHz, 103.88 ± 3.95%, n = 9; *p* = 0.0477, unpaired *t*-test). **Middle**, ITDP was not occluded in slices prepared from conditioned rats with white noise (naïve, 128.24 ± 7.62%, n = 8; white noise, 121.56 ± 8.47%, n = 9; *p* = 0.5705, unpaired *t*-test). **Right**, ITDP was occluded in slices prepared from conditioned rats with the FM tone (naïve, 121.22 ± 3.79%, n = 7; FM tone, 91.36 ± 3.67%, n = 5; *p* = 0.0003, unpaired *t*-test; scale bar, 10 ms and 50 pA). (**C**) Poisson LTP was occluded in slices prepared from rats conditioned only with white noise but not with the other two auditory CS. **Left**, ITDP was not occluded in slices prepared from conditioned rats with 2.8 kHz (naïve, 134.38 ± 14.18%, n = 8; 2.8 kHz, 146.60 ± 17.19%, n = 6; *p* = 0.5907, unpaired *t*-test). **Middle**, ITDP was occluded in slices prepared from conditioned rats with white noise (naïve, 138.84 ± 11.45%, n = 6; white noise, 108.49 ± 4.53%, n = 8; *p* = 0.0182, unpaired *t*-test). **Right**, ITDP was not occluded in slices prepared from conditioned rats with the FM tone (naïve, 137.67 ± 9.24%, n = 5; FM tone, 153.17 ± 13.74%, n = 7; *p* = 0.4141, unpaired *t*-test; scale bar, 50 ms and 3 mV).

In the C-LA pathway, we observed a different pattern of LTP occlusion (Fig. 3). Pairing LTP was occluded in slices prepared from conditioned rats with white noise and the FM tone, respectively, but not with the 2.8 kHz tone (naïve, 151.20 ± 11.46%, n = 11, 2.8 kHz, 155.64 ± 12.72%, n = 8, *p* = 0.8002, unpaired *t*-test; naïve, 149.44 ± 11.58%, n = 6, white noise, 115.30 ± 6.86%, n = 6, *p* = 0.0294, unpaired *t*-test; naïve, 133.36 ± 10.02%, n = 8; FM tone, 105.99 ± 5.25%, n = 9, *p* = 0.0244, unpaired *t*-test) (Fig. 3A). ITDP was occluded in slices prepared from conditioned rats with the 2.8 kHz and FM tone, respectively, but not with white noise (naïve, 130.38 ± 8.92%, n = 17, 2.8 kHz, 103.88 ± 3.95%, n = 9, *p* = 0.0477, unpaired *t*-test; naïve noise, 128.24 ± 7.62%, n = 8; white noise, 121.56 ± 8.47%, n = 9, *p* = 0.5705, unpaired *t*-test; naïve, 121.22 ± 3.79%, n = 7, FM tone, 91.36 ± 3.67%, n = 5, *p* = 0.0003, unpaired *t*-test) (Fig. 3B). Poisson LTP was occluded in slices prepared from conditioned rats only with white noise but not with the other two auditory CSs (naïve, 134.38 ± 14.18%, n = 8, 2.8 kHz, 146.60 ± 17.19%, n = 6, *p* = 0.5907, unpaired *t*-test; naïve, 138.84 ± 11.45%, n = 6, white noise, 108.49 ± 4.53%, n = 8, *p* = 0.0182, unpaired *t*-test; naïve, 137.67 ± 9.24%, n = 5, FM tone, 153.17 ± 13.74%, n = 7; *p* = 0.4141, unpaired *t*-test) (Fig. 3C). Taken together, these data suggest that fear conditioning with different auditory CSs utilizes a distinct set of LA LTP mechanisms, respectively; that is, (1) T-LA pairing LTP and C-LA ITDP for 2.8 kHz tone; (2) T-LA ITDP, C-LA pairing-induced LTP and C-LA Poisson LTP for white noise; and (3) T-LA pairing LTP and C-LA pairing LTP and C-LA ITDP for the FM tone.

### Fear conditioning with the different auditory CSs induces CP-AMPAR insertion that persists over variable periods

One of the representative plastic changes that occur after fear conditioning is transient synaptic insertion of CP-AMPARs at T-LA synapses^27^,^34^. We therefore questioned whether conditioning with the three auditory CSs produces differential effects on CP-AMPAR insertion, respectively. Unlike in prior experiments (in which synaptic changes and LTP were measured) that required strong conditioning to recruit as many synapses as possible, we chose to use three CS-US pairings because additional pairings in following days may alter CP-AMPAR insertion, as shown in a previous study^34^. Additionally, we prepared brain slices 6 hr after conditioning because in rats, CP-AMPAR insertion peaks and fear memory become consolidated at this time point^33^,^34^.

**Figure 4:**
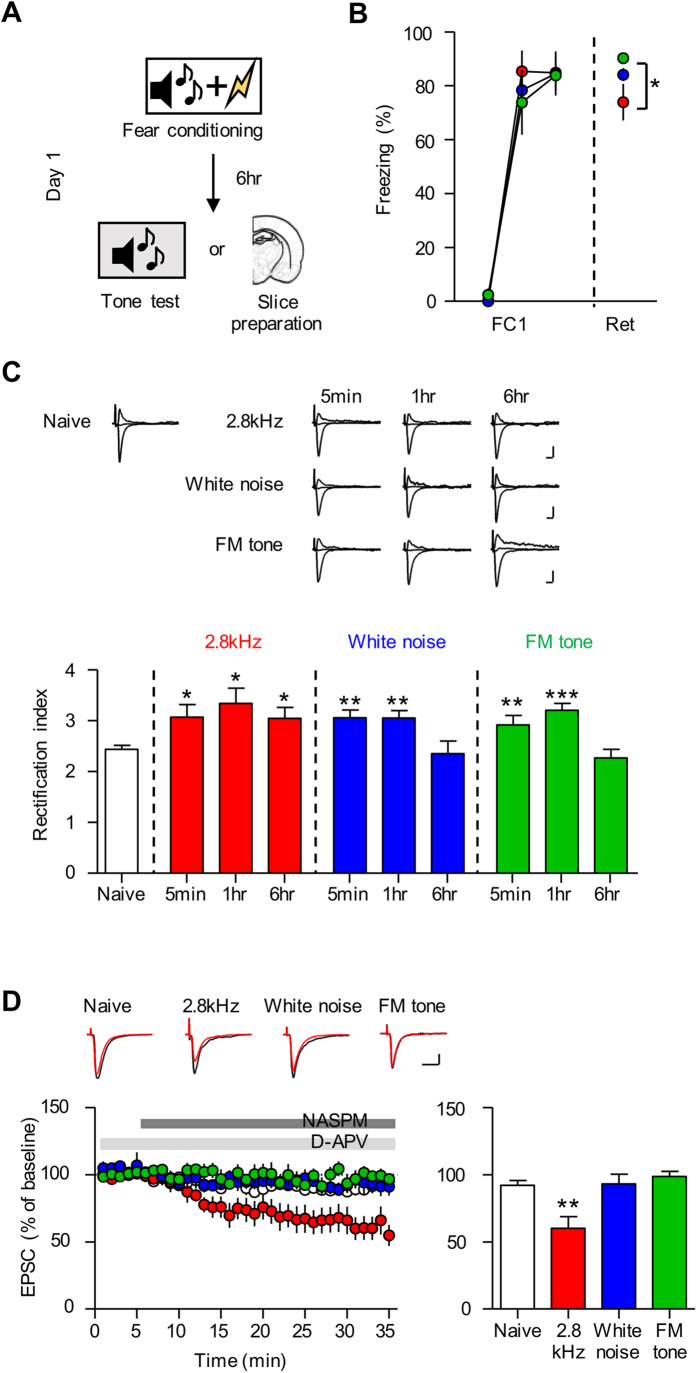
Fear conditioning with different auditory CS induces CP-AMPAR insertion that persists over variable periods. (**A**) The behavioral procedure is shown. Acute brain slices were prepared at three time points (5 min, 1 hr, and 6 hr) after conditioning. (**B**) Freezing responses are shown during fear conditioning and subsequent memory retention periods (6 hr after fear conditioning). Retention of conditioned fear memory in the FM tone group but not in the white noise group, was significantly larger than that in the 2.8 kHz group (red circle, 2.8 kHz, 73.89 ± 6.79%, n = 5; blue circle, white noise, 84.11 ± 2.37%, n = 5; green circle, FM tone, 90.15 ± 2.12%, n = 7; F_(2,16)_ = 4.430, *p* = 0.0323, one-way ANOVA; *p < 0.05, Newman-Keuls post-hoc test). (**C)** RI in the T-LA pathway was enhanced in slices prepared after conditioning with the three auditory CS relative to naïve controls (*p < 0.05, **p < 0.01 and ***p < 0.001 for naïve vs. conditioned groups, one-way ANOVA followed by Newman-Keuls post-hoc test). Please note that RI was initially enhanced in all three groups, but the RI enhancement persisted up to 6 hr after conditioning only in the 2.8 kHz group (red bar, 2.8 kHz; blue bar, white noise; green bar, FM tone; scale bar, 10 ms and 50 pA). (**D**) NASPM inhibited EPSCs at T-LA synapses in slices prepared 6 hr after conditioning only with the 2.8 kHz tone but not with white noise or the FM tone (red, 2.8 kHz; blue, white noise; green, FM tone; **p < 0.01 for naïve vs. 2.8 kHz groups, one-way ANOVA followed by Newman-Keuls post-hoc test; scale bar, 20 ms and 50 pA). NASPM was applied 5 min after the start of stable recordings.

We prepared acute brain slices at three time points (5 min, 1 hr, 6 hr) after conditioning and assessed the rectification index, which correlates with CP-AMPAR insertion (Fig. 4A,B)^39^,^40^,^41^,^42^. Conditioning with one of the three CSs (2.8 kHz tone, white noise, FM tone) produced enhancements in the rectification index (RI) in slices prepared 5 min and 1 hr after conditioning (naïve, 2.44 ± 0.08, n = 17; 2.8 kHz group, 5 min, 3.07 ± 0.25, n = 13, 1 hr, 3.34 ± 0.30, n = 7, 6 hr, 3.05 ± 0.21, n = 9, F_(3,45)_ = 4.182, *p* = 0.0112, one-way ANOVA, *p* < 0.05 naïve vs. 5 min and 1 hr, Newman-Keuls post-hoc test; white noise group, 5 min, 3.06 ± 0.15, n = 11, 1 hr, 3.06 ± 0.15, n = 8, 6 hr, 2.35 ± 0.25, n = 8, F_(3.43)_ = 6.802, *p* = 0.0008, one-way ANOVA, *p* < 0.01 naïve vs. 5 min and 1 hr, Newman-Keuls post-hoc test; FM tone group, 5 min, 2.92 ± 0.19, n = 11, 1 hr, 3.21 ± 0.13, n = 11, 6 hr, 2.27 ± 0.17, n = 7, F_(3.45)_ = 9.299, *p* = 0.0001, one-way ANOVA, *p* < 0.01 naïve vs. 5 min, *p* < 0.001 naïve vs. 1 hr, Newman-Keuls post-hoc test) (Fig. 4C). By contrast, conditioning only with the 2.8 kHz tone, but not with white noise or the FM tone, produced enhancements in RI 6 hr after conditioning (p < 0.05, Newman-Keuls post-hoc test) (Fig. 4C). To confirm the RI experiment results, we utilized 1-naphthylacetylsperimine (NASPM), a specific blocker of CP-AMPARs^40^. NASPM significantly inhibited evoked EPSCs in acute brain slices 6 hr after conditioning only with the 2.8 kHz tone, but not with white noise or the FM tone, consistent with the RI experiment results (naïve, 92.00 ± 3.60%, n = 9; 2.8 kHz, 60.16 ± 8.57%, n = 4; white noise, 93.14 ± 7.19%, n = 5; FM tone, 98.53 ± 4.01%, n = 6; F_(3,23)_ = 8.075, *p* = 0.0010, One-way ANOVA; *p* < 0.01 for naïve vs. 2.8 kHz groups, Newman-Keuls post-hoc test) (Fig. 4D). To rule out the possibility that the NASPM inhibition is mediated by kainate receptors (see ref. 11), we used UBP302, a selective blocker for kainate receptors^43^, and we did not find a significant effect of UBP302 on T-LA synaptic transmission in slices prepared from naïve rats or conditioned rats with the 2.8 kHz tone (Supplementary Fig. 1).

In sum, conditioning with the 2.8 kHz tone appeared to produce persistent insertion up to 6 hr after conditioning, whereas conditioning with white noise or the FM tone seemed to produce transient insertion that is maintained less than 6 hr after conditioning. It should be stressed that in rats, conditioning with certain types of auditory CSs (white noise and 2.8 kHz tone) produces transient insertion of CP AMPARs that is removed even before the end of fear memory consolidation (<6 hr after conditioning), in marked contrast with a previous result reported for mice^27^.

### Reconsolidation-update is effective only in the 2.8 kHz group, in which synaptic CP-AMPARs are present after fear memory consolidation

**Figure 5:**
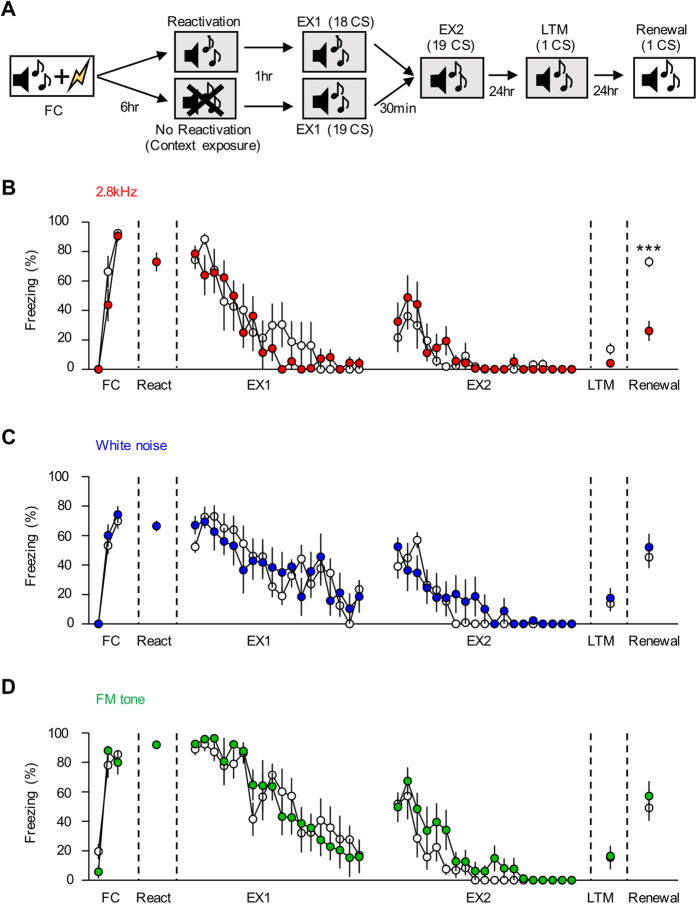
Reconsolidation-update is effective only in the 2.8 kHz group but not in the white noise or FM tone groups. (**A**) The behavioral procedure of reconsolidation-update is shown. A fear memory was reactivated 6 hr after conditioning, and extinction training was initiated 1 hr after the memory reactivation. The memory reactivation was omitted in extinction controls. We assessed long-term memory (LTM) 24 hr after the last session of extinction training and assessed fear renewal 24 hr after the LTM test. (**B**) Reconsolidation-update following 2.8 kHz tone conditioning prevented fear renewal (***p < 0.001, unpaired *t*-test). (**C,D**) Reconsolidation-update following conditioning with white noise or FM tones did not prevent fear renewal.

Synaptic insertion of CP AMPARs at LA synapses after fear conditioning is known to be critical for reconsolidation-update (a variant of extinction which produces erasure of consolidated memory), in which a fear memory is simply reactivated approximately 1 hr before extinction training^27^,^34^. We therefore asked whether reconsolidation-update would only erase memory in the group (i.e., 2.8 kHz) in which CP-AMPARs are present at LA synapses after fear memory is consolidated (i.e., 6 hr after conditioning) (Fig. 5). A fear memory was reactivated 6 hr after conditioning, and extinction training was initiated 1 hr after the memory reactivation. The memory reactivation was omitted in extinction controls. We assessed long-term memory (LTM) 24 hr after the end of extinction training and tested fear renewal as a measure of memory erasure 24 hr after the LTM test. Extinction without memory reactivation in the three groups (2.8 kHz, white noise, FM tone) did not erase fear memory as successful fear renewal compared with extinction controls, whereas reconsolidation-update erased fear memory only in the 2.8 kHz group, as judged by the failure of fear renewal (reactivation, 26.10 ± 6.81%, n = 8, no reactivation, 72.88 ± 3.57%, n = 6; *p* < 0.001, unpaired t-test) (Fig. 5B). In the other two groups, successful fear renewal occurred even after reconsolidation-update compared with extinction controls, suggesting that fear memory was not erased (for white noise group, reactivation, 51.99 ± 9.26%, n = 6, no reactivation, 45.28 ± 7.50%, n = 8, *p* = 0.5799, unpaired *t*-test; for FM tone group, reactivation, 57.13 ± 10.20%, n = 6, no reactivation, 49.09 ± 8.73%, n = 5, *p* = 0.5731, unpaired *t*-test) (Fig. 5C,D). These findings are consistent with the results indicating that only the 2.8 kHz group retains synaptic CP-AMPARs after memory consolidation (6 hr after conditioning), and the lack of synaptic CP-AMPARs in the other two groups after memory consolidation may explain the failure of reconsolidation-update. Therefore, these data confirm a previous finding indicating that the presence of CP-AMPARs at LA synapses is required for reconsolidation-update and further suggest the possibility that variable experimental outcomes with reconsolidation update are caused by different tone types used as CSs, as indicated in previous studies.

## Discussion

The present study proposes a novel physiological role of synaptic plasticity; that is, distinct forms of synaptic plasticity at LA synapses are used to encode different auditory CSs, which leads to different memory characteristics. Fear conditioning with different auditory CSs recruits distinct forms of LTP at both T-LA and C-LA synapses and induces CP-AMPAR insertion that persists for a variable duration at T-LA synapses. Importantly, conditioning with a 2.8 kHz tone, but not with white noise or with an FM tone, induces CP-AMPAR insertion ≥6 hr after conditioning (at the time point when fear memory consolidation is completed; see ref. 44). Consistent with these data, reconsolidation-update, which requires the presence of synaptic CP-AMPARs, is effective only in the 2.8 kHz group but not in the other two groups. Thus, our findings provide one explanation for why many forms of synaptic plasticity exist at LA synapses. The firing patterns of pre- and postsynaptic neurons, which are unique for each type of auditory CS, may lead to induction of specific forms of LA synaptic plasticity^7^,^45^,^46^.

Differences exist in the pre/post activity pattern in response to distinct types of auditory CS at LA synapses. LA neurons receive CS-evoked excitatory inputs directly from the auditory thalamus (MGm/PIN) and auditory association cortex (TE3/PRh)^1^. LA neurons also receive US-evoked excitatory inputs from thalamic and cortical areas^47^,^48^. Therefore, distinct types of CS and US produce differential firing patterns in the brain regions that retain pre- or postsynaptic cells. The three types of auditory CS used in the present study (pure tone, white noise and FM tone) are known to induce different auditory-evoked responses (approximately 30, 10 and 30 Hz responses in the auditory association cortex, respectively^49^,^50^; approximately 30, 33 and 32 Hz responses in the auditory thalamus, respectively^51^,^52^,^53^; and approximately 10, 100 and 40 Hz responses in the LA, respectively^51^,^54^). US also induce approximately 300 Hz responses in LA neurons^55^. These differences in pre/post activity patterns in response to the three types of CS and US may contribute to differential recruitment of LTP mechanisms at LA synapses, although we do not currently have any information to predict the plasticity rules for the CS-dependent changes. It remains to be elucidated what types of plasticity can be induced with repetitive stimulation resembling *in vivo* activity patterns.

Our findings suggest that caution should be taken when using different types of CS, even CS of the same modality. Many laboratories that have studied fear memory have chosen to use one type of CS without any precaution or purpose; hence, previous results obtained using different CSs of the same modality (or even of different modalities) were considered together to formulate a specific hypothesis for fear memory processing. We recognized the inconsistent effects of reconsolidation-update on fear memory erasure in previous studies^37^,^38^. In the present study, we found that reconsolidation-update is effective in conditioning only with the 2.8 kHz tone but not with white noise or with the FM tone. Although we did not test pure tones of other frequencies, it is also possible that conditioning with pure tones of different frequencies would give rise to variable effects of reconsolidation update on memory erasure^27^,^32^,^56^,^57^. Consistent with our results, fear memory was erased by reconsolidation-update when a 5 kHz or 2 kHz tone was used as the CS^27^,^32^, but it was not erased when a 750 Hz or 10 Hz tone was used as the CS^56^,^57^.

In addition to the LA, the primary auditory cortex is well-known to exhibit CS-dependent changes that result from pairing of auditory CS and unconditioned stimuli^58^,^59^. Moreover, the CS-dependent changes in the primary auditory cortex are far more selective to detailed frequencies of auditory CS than are the changes at LA synapses. It is possible that associative fear memories that are encoded in the primary auditory cortex are explicit memories that lead to conscious awareness of danger to auditory CS with different frequencies. By contrast, associative fear memories that are encoded at LA synapses are implicit memories that respond unconsciously and reflexively to auditory CS. It is therefore speculative that distinct plasticity mechanisms at LA synapses underlying different auditory CS, as suggested in the present manuscript, retain functional advantages for individual survival. Therefore, some tones that are more critical for survival than other tones produce a more persistent form of fear memory. LA neurons have also been shown to exhibit CS-dependent changes in a previous study^10^. It was shown that 1 kHz and FM tones were used as CS and that brief presentation of either tone 3 hrs after memory retrieval produced different responses in CS-evoked field potentials in the LA; therefore, the FM tone produced no significant changes, but the 1 kHz tone induced a potentiation. Our findings in the present study can provide a new explanation for this particular observation; that is, different types of auditory CS (1 kHz and FM tones) are encoded as distinct forms of LA synaptic plasticity so that presentation of the 1 kHz or FM tone after memory retrieval can lead to differential CS-evoked responses in the LA. Therefore, different facets of fear memory appear to be encoded in distinct brain regions.

In the present study, it is not clear how distinct LTP mechanisms lead to CP-AMPAR insertion for variable periods. Our findings from the LTP experiment are especially difficult to reconcile with those from the CP-AMPAR experiment because the T-LA LTP mechanisms that were induced by 2.8 kHz and FM tones were similar; however, CP-AMPARs were inserted for different time periods in the two groups (see Fig. 2). One possibility to account for this discrepancy is that T-LA synapses have new types of LTP that can be induced differentially by the two tones. Alternatively, CP-AMPAR insertion at T-LA synapses could be induced and regulated by heterosynaptic plasticity as shown in previous studies^60^ such that CS-evoked stimulation of C-LA synapses sends diffusible messengers to T-LA synapses to insert CP-AMPARs at T-LA synapses for variable periods. Finally, we do not rule out the possibility that CP-AMPAR insertion exists at C-LA synapses (which is critical for reconsolidation update) because C-LA synapses also play a major role in maintaining fear memory^61^.

Although we only tested memory modulation in relation to CP-AMPAR insertion (reconsolidation-update) in the present study, there would be additional differences in plastic changes and memory modulation. For example, we found that the three different auditory CSs recruit distinct forms of LA LTP, some of which have been shown to have pre- and postsynaptic expression mechanisms at either T-LA or C-LA synapses^8^,^11^,^17^,^18^,^19^,^20^,^22^,^23^,^25^. Depending on the forms of recruited LTP, molecular and cellular constituents at LA synapses would be altered over the long term, which may affect subsequent memory modulation by behavioral stimuli or by drug challenges. For example, presynaptic release probability is known to be enhanced in the case of pairing LTP at C-LA synapses^8^. Furthermore, presynaptic expression has been shown to be maintained by enhanced activity of presynaptic L-type calcium channels in the case of presynaptic LTP at C-LA synapses^25^. Additionally, AMPAR trafficking is known to be critical in the maintenance of pairing LTP at T-LA synapses^17^. Therefore, conditioned fear memories to various cues would be superficially similar, but their molecular and cellular constituents may differ significantly, contributing to the maintenance and erasure of the memories. Importantly, the present findings should be considered in the development of new drugs or cognitive therapeutic regimens to treat fear memory-related disorders.

## Methods

### Subjects and behavioral procedures

Male Sprague-Dawley rats (4–6 weeks old) were obtained from a commercial supplier (Orient Bio Inc., Seongnam, Republic of Korea). Rats were given free access to food and water and housed under an inverted 12/12 hr light-dark cycle (lights off at 9:00 AM). Behavioral training was performed during the dark portion of the cycle. Animal experiments were carried out in accordance with the protocol approved by the Institute of Laboratory Animal Resources of Seoul National University.

Fear conditioning was performed in a rectangular Plexiglas chamber with a metal grid floor connected to an electrical current source (Coulbourn Instruments, Allentown, PA) in a sound-attenuating room (referred to as “context A”). In the room with white light on, the sound of a fan served as background noise. For fear conditioning, rats were placed in the conditioning chamber and left undisturbed for 2 min. One of three types of sounds was used as a CS: a pure tone (2.8 kHz, 80 dB), white noise (80 dB), or an FM tone (12.5 kHz carrier frequency, 50 Hz modulation frequency, 2.5 kHz modulation depth, 1 ms ramp, 80 dB). A CS co-terminating with an electric foot shock (1.0 mA, 1 s) was then presented three times at an average interval of 100 sec. Rats were returned to their home cages 60 s after the last shock was presented. Extinction training and retention tests were performed in a cylindrical chamber in a sound-attenuating cubicle (Med Associates Inc., Albans, VT) with a black Formica floor (referred to as “context B”). The cubicle with the light off was ventilated with a fan for background noise. A camera was mounted on top of the chambers to record behaviors.

All of the training sessions were videotaped and conditioned freezing was quantified by trained observers that were blind to the experimental groups. During the CS presentation (30 sec), the animals were considered frozen when there was no movement except for respiratory activity for more than 1 sec. The total freezing time was normalized to the duration of the CS presentation.

### Slice preparation

Brain slices were prepared using techniques previously described^62^,^63^,^64^. Rats were anesthetized with isoflurane and decapitated to remove the brain. The isolated whole brains were placed in an ice-cold modified aCSF solution containing the following (in mM): 175 sucrose, 20 NaCl, 3.5 KCl, 1.25 NaH2PO4, 26 NaHCO3, 1.3 MgCl2 and 11 D-(+)-glucose. Solutions were then bubbled with 95%O2/5% CO2. Coronal slices (300 μm) including the LA were cut using a vibroslicer (VT1200, Leica, Germany) and incubated in normal aCSF containing the following (in mM): 120 NaCl, 3.5 KCl, 1.25 NaH2PO4, 26 NaHCO3, 1.3 MgCl2, 2 CaCl2 and 11 D-(+)-glucose, bubbled at room temperature with 95% O2/5% CO2. Immediately before transferring a slice to the recording chamber, the cortex overlying the LA was cut away with a scalpel so that cortical epileptic burst discharges would not invade the LA in the presence of picrotoxin.

### Afferent stimulation

Brain slices were selected based on the presence of a well-isolated, sharply defined trunk (containing thalamic afferents) crossing the dorsolateral division of the LA and the presence of the external capsule (containing cortical afferents)^63^,^65^. A concentric bipolar electrode (CBAEC75, FHC Inc, ME) was used for the stimulation of thalamic and cortical afferents. For the stimulation of thalamic afferents, a concentric bipolar electrode was placed at the midpoint of the trunk between the internal capsules^63^. For the stimulation of cortical afferents, a concentric bipolar electrode was placed on the external capsule immediately above the dorsal tip of the LA^65^. Regions and cells of interest for all recordings in the two pathways were located beneath the midpoint of the trunk spanning the LA horizontally.

### Whole-cell patch-clamp recordings

Whole-cell recordings were made using an Axopatch 200A, 700A, or 700B amplifier (Molecular Devices, CA). Recordings were obtained using pipettes with a resistance of 3–4.5 MΩ. Recordings were made under infrared differential interference contrast (IR-DIC)-enhanced visual guidance from neurons that were three to four cell layers below the surface of 300 μm thick slices at 32.0 ± 1.0 °C. The aCSF containing picrotoxin (100 μM, Sigma-Aldrich, MO) were delivered to slices via superfusion driven by a peristaltic pump (REGLO digital Ismatec, Switzerland) at a flow rate of 1.5 mL/min. The pipette series resistance was monitored throughout the experiments. If the series resistance changed by >20%, the data were discarded. Whole-cell currents were filtered at 1 kHz, digitized at up to 20 kHz, and stored on a microcomputer (Clampex 9 or 10 software; Molecular Devices, CA). All recordings were completed within 4.5 hr after slice preparation. The cells were classified as principal neurons based on the pyramidal shape of their somata. A minor portion (<5%) of recorded neurons exhibited spontaneous EPSCs with a faster decay time and larger amplitude (>100 pA), typical characteristics of interneurons in the LA, and were excluded from analysis. To prevent bias, the experimenter was blind to the behavioral groups.

### Measurement of AMPA/NMDA ratio and paired-pulse ratio (PPR)

The animals underwent fear conditioning for 2 days; preparation of brain slices for fear retention tests was then performed 24 hr after the last session of fear conditioning. For the AMPA/NMDA ratio, stimulation was delivered at a frequency of 0.067 Hz, and recording pipettes were filled with a solution containing the following (in mM): 100 Cs-gluconate, 0.6 EGTA, 10 HEPES, 5 NaCl, 20 TEA, 4 Mg-ATP, 0.3 Na-GTP, and 3 QX314, with the pH adjusted to 7.2 with CsOH and the osmolarity adjusted to approximately 297 mmol/kg with sucrose. The AMAP/NMDA ratio was calculated as the ratio of the peak EPSC amplitude at −70 mV and the EPSC amplitude 100 ms after the stimulus artifact at +40 mV.

For the paired pulse ratio (PPR), recording pipettes were filled with a solution containing the following (in mM): 115 CsMeSO4, 20 CsCl, 10 HEPES, 2.5 MgCl2, 4 Na2-ATP, 0.4 Na-GTP, 10 Na-phosphocreatine, and 0.6 EGTA, with the pH adjusted to 7.2 with CsOH and the osmolarity adjusted to approximately 297 mmol/kg with sucrose. Paired-pulse stimulation was delivered at a frequency of 0.067 Hz at 20, 50 or 100 ms inter-stimulus intervals (ISI), and EPSCs were recorded at −70 mV in the presence of D-AP5 (50 μM; Tocris, Bristol, United Kingdom). PPR was calculated as the ratio between the peak amplitudes of the second and first EPSCs.

### LTP induction in T-LA pathway

The rats underwent fear conditioning for 2 days; brain slices were then prepared 24 hr after the last session of fear conditioning. For the induction of pairing LTP, recording pipettes were filled with a solution containing the following (in mM): 115 CsMeSO4, 20 CsCl, 10 HEPES, 2.5 MgCl2, 4 Na2-ATP, 0.4 Na-GTP, 10 Na-phosphocreatine, and 0.6 EGTA, with the pH adjusted to 7.2 with CsOH and the osmolarity adjusted to approximately 290 mmol/kg with sucrose^17^. For LTP induction, stimulation was delivered at a frequency of 3 Hz for 3 min while a postsynaptic cell was depolarized to 0 mV^17^.

For the induction of ITDP, recording pipettes were filled with a solution containing the following (in mM): 150 K-gluconate, 10 HEPES, 5 NaCl, 1 MgCl2, 2 Mg-ATP, 0.6 Na-GTP, and 0.2 EGTA, with the pH adjusted to 7.25 with KOH and the osmolarity adjusted to approximately 290 mmol/kg with sucrose^11^. The ITDP protocol consisted in the paired stimulation of the thalamic and cortical inputs for 90 s at a frequency of 1 Hz, and the stimulation of the cortical input was delivered 15 ms earlier than that of the thalamic input^11^. During presynaptic stimulation, a post-synaptic neuron was voltage-clamped at a holding potential of −70 mV.

For the induction of presynaptic LTP, recording pipettes were filled with a solution containing the following (in mM): 120 KMeSO4, 10 HEPES, 5 NaCl, 1 MgCl2, 2 Mg-ATP, 0.1 Na-GTP, and 0.2 EGTA, with the pH adjusted to 7.2 with KOH and the osmolarity adjusted to approximately 290 mmol/kg with sucrose^20^. LTP was induced by 240 paired presynaptic stimuli (with 50 ms ISI) delivered at a frequency of 2 Hz to the thalamic input at a holding potential of −70 mV^20^.

LTP induction in C-LA pathway. The rats underwent fear conditioning for 2 days; brain slices were then prepared 24 hr after the last session of fear conditioning. For the induction of pairing LTP, recording pipettes were filled with a solution containing the following (in mM): 120 K-gluconate, 5 NaCl, 1 MgCl2, 0.2 EGTA, 10 HEPES. 2 Mg-ATP, and 0.1 Na-GTP, with the pH adjusted 7.2 with KOH and the osmolarity adjusted to approximately 290 mmol/kg with sucrose^8^. LTP was induced by 80 presynaptic stimuli at a frequency of 2 Hz to the cortical input while a postsynaptic cell was held at +30 mV^8^.

For the induction of ITDP, recording pipettes were filled with a solution containing the following (in mM): 150 K-gluconate, 10 HEPES, 5 NaCl, 1 MgCl2, 2 Mg-ATP, 0.6 Na-GTP, and 0.2 EGTA, with the pH adjusted to 7.25 with KOH and the osmolarity adjusted to approximately 290 mmol kg with sucrose^11^. The ITDP protocol consisted in the paired stimulation of the thalamic and cortical inputs for 90 s at a frequency of 1 Hz, and the stimulation of the thalamic input was delivered 15 ms earlier than that of the cortical input^11^. During the presynaptic stimulation, a post-synaptic neuron was voltage-clamped at a holding potential of −70 mV.

For the induction of Poisson LTP, recording pipettes were filled with a solution containing the following (in mM): 120 K-gluconate, 20 KCl, 10 HEPES, 10 phosphocreatine, 4 Mg-ATP, 0.3 Na-GTP with the pH adjusted to 7.25 with KOH and the osmolarity adjusted to approximately 295 mmol/kg^23^. Excitatory postsynaptic potentials (EPSPs) were recorded in a current-clamp mode. For induction of LTP, thalamic and cortical afferents were randomly co-stimulated using Poisson-train stimulation (45 stimuli at an average frequency of 30 Hz)^23^.

### RI measurement and NASPM treatment

To measure CP-AMPAR activity at T-LA synapses, both biophysical (rectification index of AMPAR EPSCs) and pharmacological (sensitivity to 1-naplhthylacetyl spermine trihydrochloride (NASPM)) approaches were employed. The rats underwent fear conditioning with three pairings of CS-US. A retention test was then performed 6 hr after conditioning. Brain slices were prepared at a fixed time (4–5 PM) to avoid circadian variation of CP-AMPAR activity^66^,^67^. The whole-cell recordings were performed with the recording pipettes filled with 100 Cs-gluconate, 0.6 EGTA, 10 HEPES, 5 NaCl, 20 TEA, 4 Mg-ATP, 0.3 Na-GTP, and 3 QX314. Pipette solutions were adjusted to pH 7.2 with CsOH and to osmolarity 297 mmol/kg with sucrose. Neurons were voltage-clamped at −70 mV, and stimulation was delivered to the thalamic input at a frequency of 0.065 Hz.

To estimate RI, brain slices were prepared 5 min, 1 hr or 6 hr after conditioning, and spermine (0.1 mM; Tocris, Bristol, United Kingdom) was added to the internal solution. RI was calculated as the ratio of the amplitudes: EPSChyperpolarized/EPSCdepolarized. That is, RI was calculated by dividing the absolute amplitude of the average EPSC measured at −70 mV by that at +40 mV, assuming a reversal potential (Erev) of 0 mV^68^. The reversal potential (Erev) did not vary significantly among the different groups (Naïve, 14.88 ± 0.26 mV, n = 17; 2.8 kHz (5 min), 15.73 ± 0.35 mV, n = 13; 2.8 kHz (1 hr), 14.86 ± 0.63 mV, n = 7; 2.8 kHz (6 hr), 15.67 ± 0.48 mV, n = 9; white noise (5 min), 14.91 ± 0.49 mV, n = 11; white noise (1 hr), 15.56 ± 0.26 mV, n = 8; white noise (6 hr), 14.50 ± 0.60 mV, n = 8; FM tone (5 min), 14.09 ± 0.59 mV, n = 11; FM tone (1 hr), 15.09 ± 0.46, n = 11; FM tone (6 hr), 14.57 ± 0.75 mV, n = 7; F(7.86) = 1.568, p = 0.1572, one-way ANOVA; p > 0.05 for all pairs, Newman-Keuls post-hoc test). This method is based on the fact that AMPA receptors lacking GluA2 subunits display inward rectification due to the voltage-dependent block of the channel pores by polyamines at positive membrane potentials^12^. The recordings were started 20 min after the onset of whole-cell configuration to ensure sufficient diffusion of the exogenous spermine into the cell interior. D-AP5 (50 μM; Tocris, Bristol, United Kingdom) was used to isolate AMPAR-mediated EPSCs at positive potentials.

To measure the sensitivity to NASPM, brain slices were prepared 6 hr after conditioning, and AMPAR-mediated EPSCs were monitored before, during and after the application of NASPM (50 μM, Sigma-Aldrich, MO) in the presence of D-AP5 (50 μM, Tocris, Bristol, United Kingdom). The sensitivity to NASPM was estimated by the percent inhibition by NASPM (averaged EPSC amplitude for the last 5 min of the whole recording divided by averaged EPSC amplitude for 5 min of baseline).

### Reconsolidation-update

The rats underwent fear conditioning with three pairings of CS-US (context A). They received either an exposure to one CS in a different context (context B) or to the context alone 6 hr after conditioning. The rats were then stored in their home cage for 1 hr. Subsequently, they received an extinction training session (EX1) consisting of 18 or 19 CSs presentations with an intertrial interval between CSs of 180 s (in context B). Rats in the reactivation group received 18 CSs, and rats in the no-reactivation group received 19 CSs such that the two groups could receive the same number of CSs. The rats were then stored in their home cage for 0.5 hr. Subsequently, they received additional extinction training session (EX2) consisting of 19 CSs presentation. Twenty-four hours after the last session of extinction training, a retention test was performed to assess LTM (in context B). Twenty-four hours after the retention test, fear renewal was tested in the conditioning context (context A).

### Statistical analysis

Sample size was determined post hoc on the basis of those use in our previous studies^63^,^65^,^68^. Subjects were randomly assigned to the experimental groups by a person who was not involve in the experiments. Data distribution was assumed to be normal but it was not formally tested. Comparisons of data among three or more groups were made using a one-way ANOVA with subsequent Newman-Keuls post hoc comparison (Figs 1, 2C and 4). The comparisons between two groups were made using a two-side unpaired t-test (Figs 2A,B, 3 and 5). p < 0.05 was considered indicative of statistical significance. All values are expressed as mean ± SEM. The data from each neuron and slice were treated as independent samples. In all experiments with behaviorally trained rats, the data included samples from three or more rats.

## Additional Information

**How to cite this article**: Park, S. *et al*. Sound tuning of amygdala plasticity in auditory fear conditioning. *Sci. Rep.* **6**, 31069; doi: 10.1038/srep31069 (2016).

## Supplementary Material

Supplementary Information
