# Supplementary material for: Sound tuning of amygdala plasticity in auditory fear conditioning
Source: Sci Rep. 2016 Aug 4;6:31069. doi: 10.1038/srep31069 (PMC4973267; doi:10.1038/srep31069)
Supplement: Supplementary Information [file srep31069-s1.pdf]

Sound tuning of amygdala plasticity in auditory fear conditioning

Authors and Affiliations

Sungmo Park[1#], Junuk Lee[1#], Kyungjoon Park[1#], Jeongyeon Kim[2], Beomjong Song[3], Ingie Hong[4], Jieun Kim[5,6], Sukwon Lee[7]*, Sukwoo Choi[1]*

[1]School of Biological Sciences, College of Natural Sciences, Seoul National University, 1 Gwanangno, Seoul 08826, Korea

[2] Center for Neuroscience and Center for Functional Connectomics, Korea Institute of Science and Technology, Seoul 136791, Korea

[3] Institute of Neuroscience, Technical University of Munich, 80333, Germany

[4] The Solomon H. Snyder Department of Neuroscience, Johns Hopkins University School of Medicine, Baltimore, Maryland 21205, USA

[5]Ewha Brain Institute, Ewha W. University, Seoul, Korea.

[6]Department of Brain and Cognitive Sciences, Scranton College, Ewha W. University, Seoul, Korea.

[7]Department of Neural Development and Disease, Korea Brain Research Institute, Daegu, Korea.

[#] These authors equally contributed to the present MS

* Correspondence to Dr. Sukwon Lee or Dr. Sukwoo Choi

E-mail: tincircle@gmail.com or sukwoo12@snu.ac.kr

Supplementary Figure 1

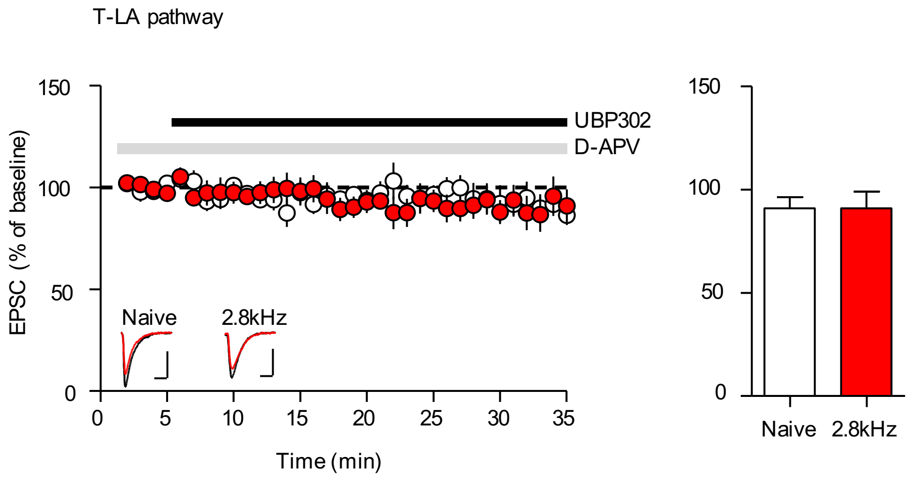

Supplementary Figure 1. Fear conditioning with the 2.8 kHz tone did not alter kainate receptor-mediated EPSCs at T-LA synapses. UBP302, a selective blocker for kainate receptors, had no significant effects on T-LA synaptic transmission in slices prepared from conditioned rats with the 2.8 kHz tone 6 hr after conditioning (naïve, 90.92 ± 5.41 %, n = 5, 2.8 kHz, 90.97 ± 8.10 %, n = 5; p = 0.9964, unpaired t-test; scale bar, 10 ms and 50 pA).
